# Peripapillary Choroidal Vascularity and Visual Correlates in Non-Arteritic Anterior Ischemic Optic Neuropathy Using Swept-Source Optical Coherence Tomography

**DOI:** 10.3389/fopht.2022.848040

**Published:** 2022-03-03

**Authors:** Edward S. Lu, Raviv Katz, John B. Miller, Eric D. Gaier

**Affiliations:** 1 Department of Ophthalmology, Massachusetts Eye and Ear, Boston, MA, United States; 2 Department of Ophthalmology, Harvard Medical School, Boston, MA, United States; 3 Harvard Retinal Imaging Lab, Massachusetts Eye and Ear, Boston, MA, United States; 4 Department of Ophthalmology, Boston Children’s Hospital, Boston, MA, United States; 5 Picower Institute for Learning and Memory, Massachusetts Institute of Technology, Cambridge, MA, United States

**Keywords:** non-arteritic anterior ischemic optic neuropathy (NAION), peripapillary choroidal thickness, peripapillary choroidal vascularity, swept-source OCT (SS-OCT), visual correlation

## Abstract

**Introduction:**

The peripapillary choroid shares a blood supply with and is directly apposed to the optic nerve, and therefore may contribute to the pathogenesis of non-arteritic anterior ischemic optic neuropathy (NAION). Prior studies evaluating peripapillary choroidal thickness (PCT) or choroidal vascularity index (CVI; the ratio of the perfused area to total choroid area) have produced mixed results. None investigated the relationship between PCT and CVI or demonstrated functional correlates. We hypothesized that greater PCT and lower CVI would correlate with visual function in patients presenting with NAION.

**Methods:**

Seventeen eyes with NAION (9 acute, 8 non-acute) and 6 unaffected “fellow” eyes in 13 patients, and 18 eyes in 18 age-matched control subjects were imaged using swept-source optical coherence tomography (SS-OCT) prospectively between 2017–2018. Mean PCT and CVI measurements were compared across groups and with respect to corresponding automated perimetric performance at the same visit.

**Results:**

Analysis of variance showed significantly greater PCT (NAION: 278 ± 65 μm, Fellow: 221 ± 50 μm, Control: 158 ± 27 μm, *p*<0.001) and lower CVI (NAION: 0.35 ± 0.03, Fellow: 0.35 ± 0.04, Control: 0.38 ± 0.02, *p*<0.005) in patients with NAION compared to control subjects. Bonferroni-corrected pairwise comparisons showed greater PCT and lower CVI in NAION-affected eyes compared to control eyes (*p* values<0.008), and no significant differences in PCT or CVI between NAION and fellow eyes (*p* values>0.06). PCT was negatively correlated with CVI among unaffected fellow eyes (r=−0.8, *p*<0.05), but not among acute NAION eyes (r=−0.1, *p*>0.7), non-acute NAION eyes (r=0.1, *p*>0.7), or controls (r=−0.3, *p*>0.2). Nasal CVI was positively correlated with mean deviation scores in non-acute NAION (r=0.8, *p*<0.02), but not among fellow unaffected eyes (r=0.8, *p*>0.05) or acutely affected NAION eyes (r=−0.3, *p*>0.4). Mean and temporal PCT correlated with pattern standard deviation scores among unaffected fellow eyes (r=0.8, *p*<0.04; r=0.9, *p*<0.03), but not among acute NAION eyes (r=−0.2, p>0.5; r=−0.1, *p*>0.7) or non-acute NAION eyes (r=0.1, *p*>0.7; r=0.05, *p*>0.9).

**Conclusion:**

NAION and unaffected fellow eyes demonstrate increased choroidal thicknesses and reduced vascular density. Perimetric performance is directly associated with vascular density among non-acutely affected eyes with NAION. Ongoing work will provide further insights into these structure-function relationships with pathogenic and pathophysiologic relevance.

## INTRODUCTION

Non-arteritic anterior ischemic optic neuropathy (NAION) is the most common acute optic neuropathy in adults over age 50 and causes sudden, painless unilateral vision loss. Patients with NAION experience long-term visual impairment and visual field deficits. The pathogenesis of NAION is not well understood but may involve vascular insufficiency to the retrolaminar optic nerve, which is supplied by the short posterior ciliary arteries (1). A small, crowded disc (“disc at risk”) may directly convey an anatomic vulnerability to vascular insufficiency that precipitates NAION (2).

The low-resistance peripapillary choroid shares a blood supply with and is directly apposed to the optic nerve, and therefore may contribute to the pathogenesis of NAION (3). Prior OCT studies evaluating peripapillary choroidal thickness (PCT) and choroidal vascular index (CVI; the ratio of the vascular area to total choroid area) have produced mixed results (3–9). Four studies have shown increased PCT in NAION eyes compared to control eyes (3, 5, 6, 9), one study found decreased PCT in NAION (4), and one study reported no difference (8). With regards to choroidal vascularity, one study found decreased CVI in NAION (7). Notably, no prior study has investigated the potential association between PCT and CVI. This relationship may be important because PCT is not an ideal surrogate for vascular supply, as it captures stromal and interstitial components in addition to the vasculature. Furthermore, no study has demonstrated visual function correlates of PCT or CVI differences.

Swept-source optical coherence tomography (SS-OCT) utilizes a faster scanning speed and longer-wavelength laser compared to spectral-domain OCT (SD-OCT) devices, thereby providing higher resolution imaging of the choroid (10). We aimed to compare PCT and CVI between NAION eyes, unaffected “fellow” eyes, and healthy control eyes. We hypothesized that SS-OCT would reveal greater PCT and lower CVI among NAION eyes that correlate with visual function. In addition, we hypothesized that choroidal thickness may be inversely associated with vascularity if both structural and vascular changes are involved in NAION pathogenesis.

## MATERIALS AND METHODS

### Participants

The prospective, observational study was approved by the institutional review board of Massachusetts Eye and Ear, and informed consent was obtained from all participants. All procedures adhered to the tenets of the Declaration of Helsinki and Health Insurance Portability and accountability Act regulations.

Adults with a clinical diagnosis of acute or non-acute NAION in one or both eyes, unaffected fellow eyes, and age-matched controls (closest match by age within 2 years) were imaged between October 2017 and December 2018. Consecutive patients with NAION presenting to protocol providers during the study period were identified, screened, and enrolled through the Neuro-ophthalmology service. Only patients with an unambiguous diagnosis of NAION assigned by an experienced neuro-ophthalmologist were included. Clinical features of NAION include sudden, painless, unilateral vision loss, relative afferent pupillary defect, optic disc edema on fundoscopy, visual field defects consistent with NAION, normal ESR/CRP levels, no signs or symptoms of giant cell arteritis, and resolution of disc edema within 2 months. Acute NAION was defined by disc edema in eyes within 3 months since onset of visual loss. Two patients with acute NAION were subsequently enrolled in a randomized, double-masked clinical trial of QPI-1007 (NCT02341560). No patients received any intervention or treatment for NAION prior to imaging and functional visual assessments. No other patients received treatment for the acute NAION episode. Control eyes were fellow eyes of patients with history of unilateral retinal detachment enrolled through the Retina service. Exclusion criteria included image quality index <30 (range 0–100) according to the device’s default settings, glaucoma, and concomitant chorioretinal or neurologic disease.

### Study Protocol

All participants underwent a complete ophthalmic examination, including Snellen best-corrected visual acuity (BCVA) measurement, slit-lamp examination, intraocular pressure (IOP) measurement, and dilated fundus examination. Participants were imaged using a SS-OCT (DRI Triton, Topcon, Japan) that uses a laser at a central wavelength of 1,050 nm and scanning speed of 100 kHz. The 12- × 9-mm 3D Volume scan protocol (“3D Wide”) was used with automated segmentation. Automated perimetry (Humphrey, 24–2 SITA standard) was performed on the day of OCT analysis in all cases. Perimetric data were not included if the reliability parameters provided by the test paradigm exceeded 30% fixation losses, 20% false positives, or 20% false negatives; however, SS-OCT data were still included for assessment of PCT and CVI. Snellen acuities were converted to logMAR for statistical analyses.

### Quantitative Analysis of Images

**FIGURE 1 |:**
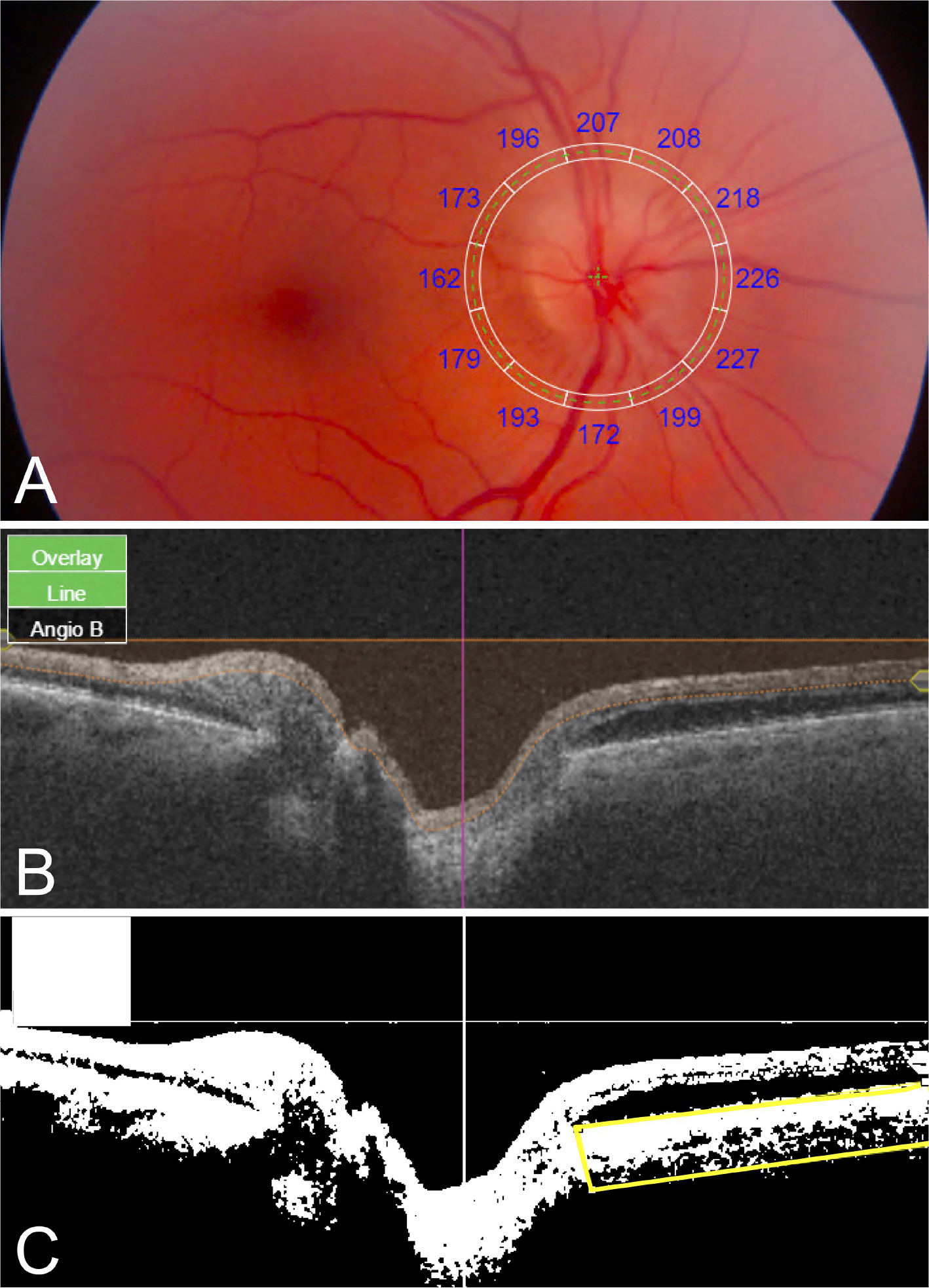
Quantitative PCT and CVI measurements using SS-OCT 12- × 9-mm images. **(A)** Fundus image with superimposed grid with sectoral PCT values. **(B)** Original SS-OCT B-scan. Bi**(C)** narized SS-OCT B-scan image with manually segmented choroid (yellow box); CVI was calculated as luminal area/total area black, luminal area; white, stromal/interstitial area.

Topcon IMAGEnet 6 software was used to obtain PCT values. For each fundus image, a superimposed 3.4-mm diameter RNFL-12 grid displaying PCT values for each of 12 peripapillary sectors was manually centered on the optic nerve (Figure 1A). For each SS-OCT B-scan, manual segmentation of the choroid-scleral interface was performed to correct segmentation errors. IMAGEnet 6 necessitates investigator viewing of the fundus image for centration, revealing the appearance of the optic disc to the investigator; thus, while manual segmentation was performed without regard for eye/patient group, it could not be performed in a masked fashion. Superior, inferior, nasal, and temporal quadrant PCTs were calculated by averaging the 3 peripapillary sectors in the corresponding quadrant. Mean PCT was calculated as the average of the 12 sectoral PCT values.

ImageJ/Fiji software (National Institutes of Health, Bethesda, MD) was used to determine CVI in the peripapillary area extending 6mm radially from the optic nerve. SS-OCT B-scans were downloaded from IMAGEnet 6. Using an adapted CVI protocol (11), original B-scan images (Figure 1B) were binarized, the peripapillary choroid was manually segmented using the polygonal selection tool, and the segmented ROI was isolated (Figure 1C). CVI was calculated as the luminal area as a proportion of the total area (luminal area plus stromal area). For each patient, one horizontal B-scan centered on the optic disc was used to calculate nasal and temporal CVI. Mean CVI was calculated as the average of nasal and temporal CVI values.

### Statistical Methods

Statistical analyses were performed using R software (R Foundation for Statistical Computing). Analysis of variance was used to determine the effect of group (NAION eyes, fellow eyes, control eyes) on PCT and CVI. Bonferroni-corrected pairwise comparisons were used to compare PCT and CVI between groups and adjust for multiple comparisons. Pearson correlations were used to determine the relationship between PCT and CVI within groups, as well as the relationship between structural (PCT, CVI) and corresponding automated perimetry performance at the same visit (mean deviation, pattern standard deviation). All statistical tests were 2-sided, and *p* values <0.05 were considered statistically significant.

## RESULTS

A total of 17 NAION eyes (9 acute, 8 non-acute) and 6 unaffected fellow eyes of 13 patients, and 18 eyes of 18 age-matched control subjects were included. Three affected eyes of patients with NAION were excluded due to poor image quality or artifacts. Patients with NAION were 62.8 ± 9.4 years of age on average (± SD) and presented with a mean BCVA of 0.12 ± 0.3 logMAR (Snellen 20/25) and IOP of 15.0 ± 2.2 mmHg (Table 1). Among the 8 non-acute eyes with NAION, the mean duration after the acute episode was 2.0 ± 2.5 years. Control participants had a mean age of 60.5 ± 5.5 years, mean BCVA of 0.02 ± 0.10 logMAR, and mean IOP of 15.8 ± 3.0 mmHg. Pairwise comparisons demonstrated no statistically significant differences in age, sex, BCVA, and IOP across groups (*p* values>0.3).

**FIGURE 2 |:**
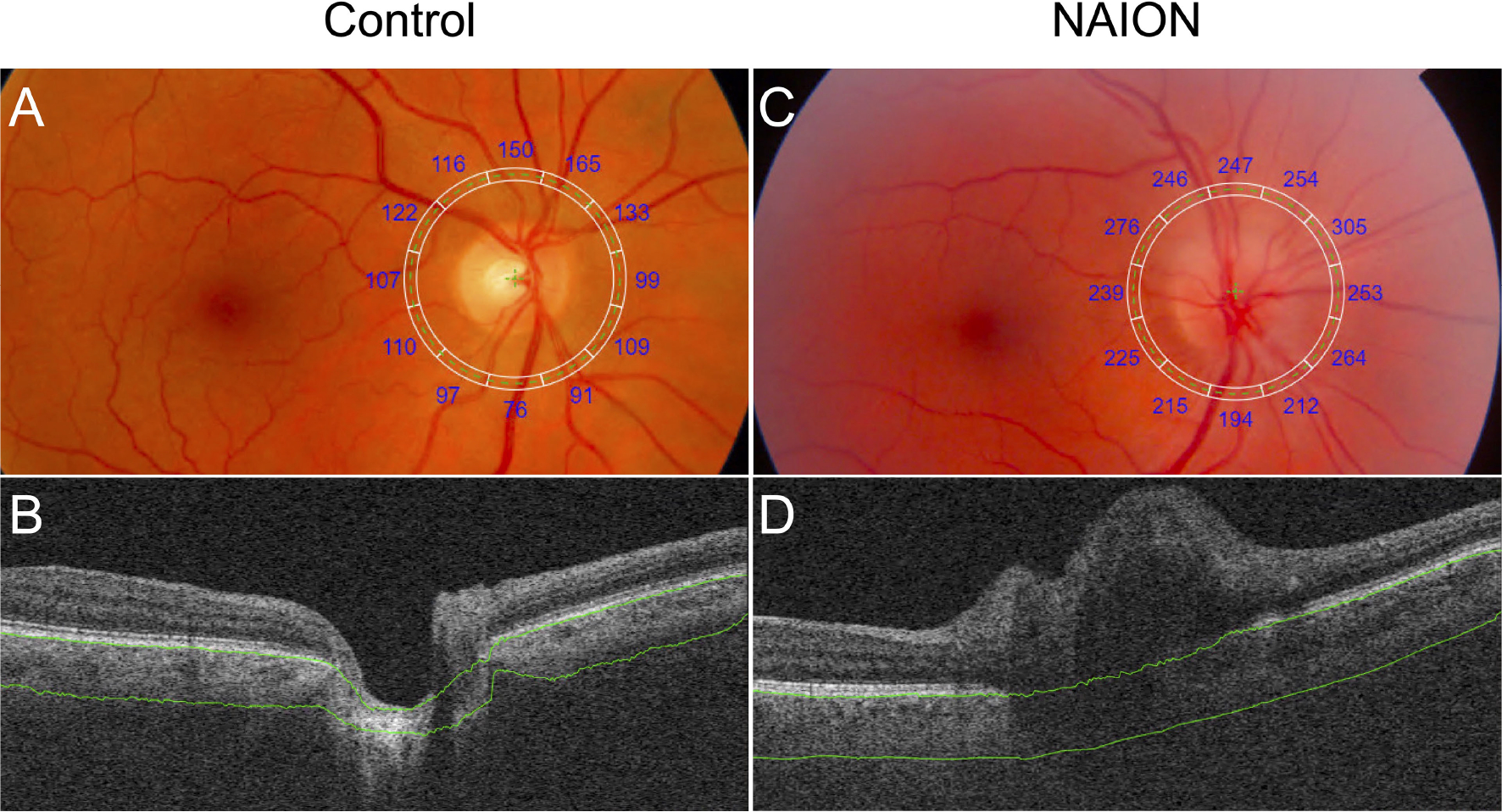
Comparison of PCT in a control eye and an eye with NAION. **(A)** Fundus image with superimposed grid with sectoral PCT values **(A)** and SS-OCT B-scan **(B)** of a control eye show decreased PCT compared to the fundus image **(C)** and SS-OCT B-scan **(D)** of an eye with NAION. Segmentation of Bruch’s membrane and the choroid-sclera interface are shown in green.

**FIGURE 3 |:**
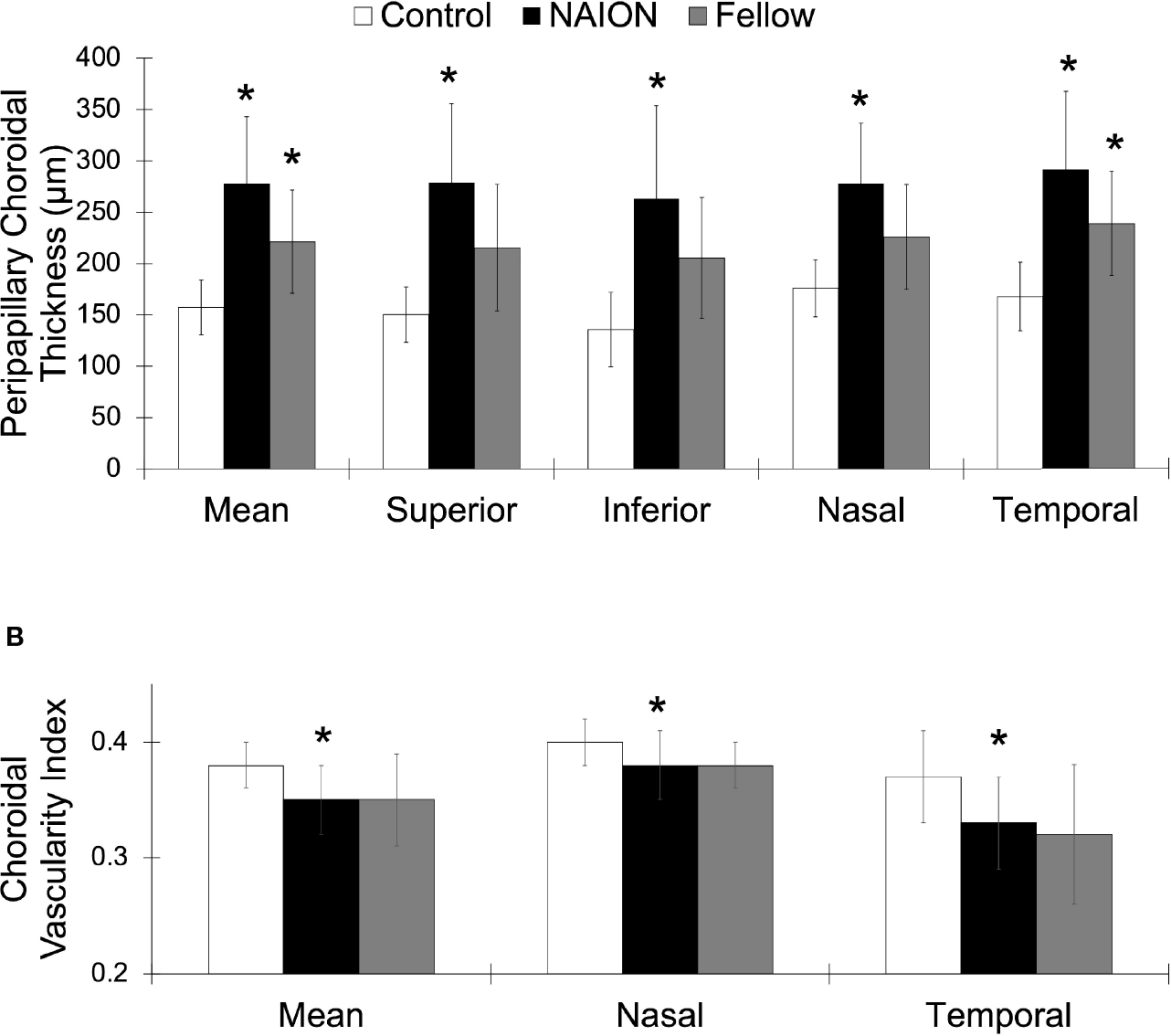
Greater PCT and lower CVI in NAION compared to control eyes. **(A)** Mean and sectoral PCT values. **(B)** Mean, nasal, and temporal CVI values. NAION (white), fellow eye (black), and control (gray) groups. *Signifies *p*<0.05 compared to control group.

No difference in PCT was found between acute and non-acute eyes with NAION (*p*>0.07). Analysis of variance showed significantly increased PCT in patients with NAION compared to control subjects (NAION: 278 ± 65 μm, Fellow: 221 ± 50 μm, Control: 158 ± 27 μm, *p*<0.001) (Figure 2). In addition, the NAION group demonstrated decreased CVI compared to controls (NAION: 0.35 ± 0.03 μm, Fellow: 0.35 ± 0.04 μm, Control: 0.38 ± 0.02 μm, *p*<0.005). Bonferroni-corrected pairwise comparisons revealed that increased PCT was present in all quadrants (superior, inferior, nasal, temporal) in affected NAION eyes compared to controls (*p* values<0.001) (Table 1 and Figure 3A). Unaffected, fellow eyes also demonstrated increased mean and temporal PCT compared to controls (*p* values<0.04); this difference was not statistically significant for the superior, inferior, and nasal quadrants (*p* values>0.06). No differences in PCT were observed between NAION and fellow eyes (*p* values>0.06).

NAION eyes exhibited decreased mean CVI compared to controls (*p*<0.008) (Table 1 and Figure 3B). Sectoral analyses revealed this difference in both nasal and temporal quadrants (*p* values<0.05). Comparing CVI measures of fellow eyes to those of controls yielded margins that bordered on statistical significance (*p* values>0.06). CVIs of NAION and fellow eyes were similar (*p* values>0.9).

**FIGURE 4 |:**
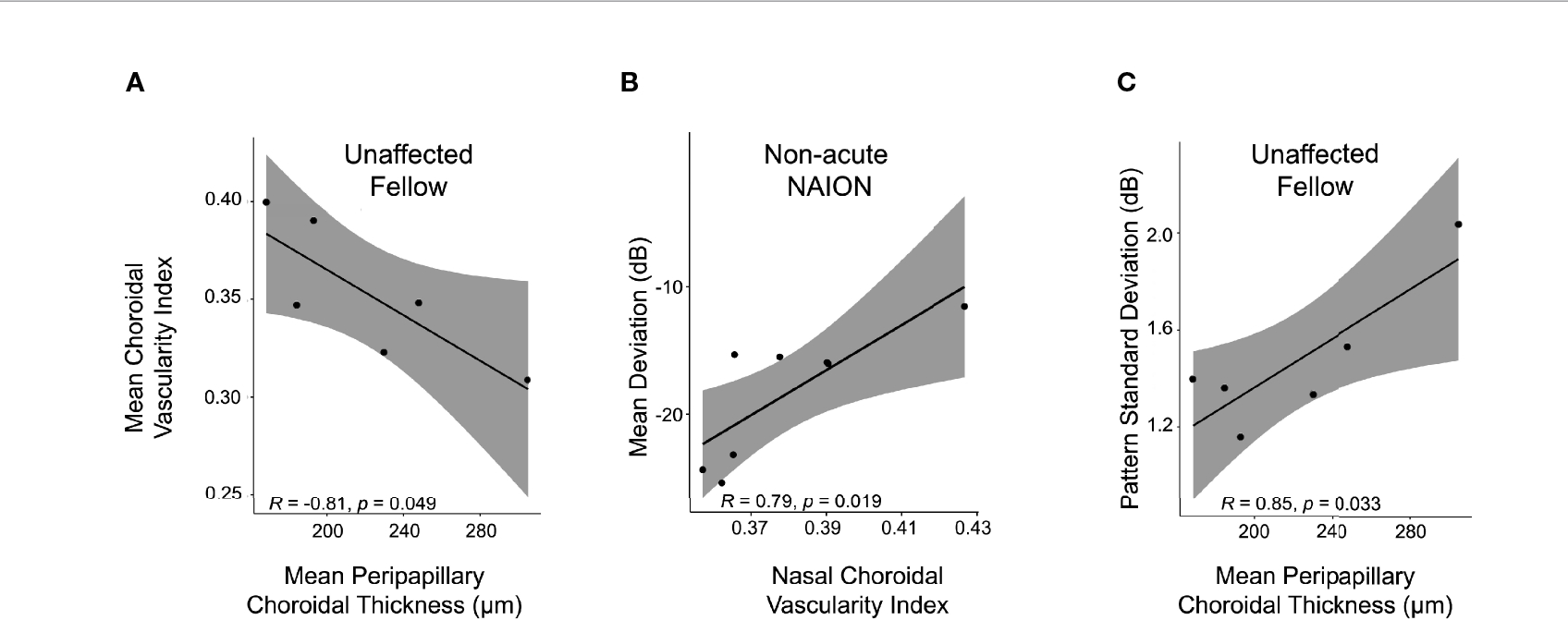
PCT/CVI relationship and structure-function correlate in NAION. **(A)** PCT correlates inversely with CVI in unaffected fellow eyes. Higher PCT corresponded with lower CVI among unaffected fellow eyes (r=−0.8, *p*<0.05), but not among acute NAION eyes (r=−0.1, *p*>0.7), non-acute NAION eyes (r=0.1, *p*>0.7) or controls (r=−0.3, *p*>0.2). **(B)** Nasal CVI correlated directly with perimetric performance in non-acute NAION (r=0.8, *p*<0.02), but not in acute NAION (r=−0.3) or fellow eyes (r=0.8, *p* values ≥0.06). **(C)** Mean PCT correlated directly with pattern standard deviation scores among unaffected fellow eyes (r=0.8, *p*<0.04), but not among acute NAION eyes (r=−0.2, *p*>0.5) or non-acute NAION eyes (r=0.1, *p*>0.7).

Mean PCT negatively correlated with mean CVI among unaffected fellow eyes (r=−0.8, *p*<0.05) (Figure 4A), but not among acute NAION eyes (r=−0.1, *p*>0.7), non-acute NAION eyes (r=0.1, *p*>0.7), or controls (r=−0.3, *p*>0.2).

We next evaluated for structure-function relationships through analysis of perimetric data. Nasal CVI was positively correlated with mean deviation scores among non-acute NAION eyes (r=0.8, *p*<0.02), but not among acute NAION eyes (r=−0.3, *p*>0.4) (Figure 4B). The nasal CVI-mean deviation relationship among unaffected eyes bordered on statistical significance (r=0.8, *p*<0.06). Mean PCT correlated with pattern standard deviation scores among unaffected fellow eyes (r=0.8, *p*<0.04) (Figure 4C), but not among acute NAION eyes (r=−0.2, *p*>0.5) or non-acute NAION eyes (r=0.1, *p*>0.7). Similarly, temporal PCT correlated with pattern standard deviation scores among unaffected fellow eyes (r=0.9, *p*<0.03), but not among acute NAION eyes (r=−0.1, *p*>0.7) or non-acute NAION eyes (r=0.05, *p*>0.9).

## DISCUSSION

We found thicker peripapillary choroids and reduced vascularity in eyes affected by NAION, and greater vascularity corresponded with relatively better perimetric performance in non-acute NAION eyes. To our knowledge, this is the first study to assess the relationship between PCT and CVI, and to identify a potential choroidal vascular structure-function correlate in NAION. Thus, these data signify important anatomic factors identifiable with OCT that may carry prognostic implications in NAION.

Several previous studies have investigated PCT in NAION using OCT (Table 2). Using the Cirrus SD-OCT, García-Basterra reported decreased PCT in 37 non-acute NAION eyes compared to healthy controls (4). Using the extended depth of imaging SD-OCT (Spectralis, Heidelberg), three groups found increased PCT in NAION and one study reported no difference in PCT between NAION and control eyes (3, 5, 8, 9). With the Triton SS-OCT (used in this study), Pérez-Sarriegui observed greater PCT whereas Guduru reported decreased CVI (6, 7). This study is consistent with the majority of prior studies that found thicker choroids and reduced vascularity in NAION using OCT. The conflicting results on PCT may be contributed by a number of factors. Studies using SD-OCT relied on manual as opposed to automated segmentation, and SS-OCT has shown greater reproducibility in measuring choroidal thickness (12, 13). In addition, SS-OCT and SD-OCT have demonstrated differences in choroidal thickness measurements that limit comparability across devices (14, 15). Moreover, systematic differences in the control groups may also contribute to the discrepancies in PCT findings.

Structural SS-OCT findings reported herein may help elucidate the poorly understood pathogenesis of NAION in the context of previously posited mechanisms. Under the compressive or compartment syndrome theory, a thicker choroid may anatomically restrict the limited optic disc space in structurally crowded nerves and thus contribute to NAION (16, 17). More specifically, a thickened choroid may compress the prelaminar neural tissues to a point that exceeds capillary perfusion pressure, resulting in a positive feedback loop of ischemia and edema that extends to the laminar and retrolaminar space (3, 18, 19). Our results and others’ indicating a greater PCT in NAION are consistent with this hypothesis. Notably, unaffected fellow eyes in patients with NAION had increased PCTs compared to controls, though this difference was restricted to mean and temporal quadrant PCTs in this study. A thicker choroid in the fellow eye of NAION patients supports an underlying structural predisposition, as fellow eyes are at significant risk of developing NAION (20).The fact that we found more prominent differences in the affected eye (and difference between affected and fellow eyes that bordered on statistical significance) may reflect why that eye was affected first. Another proposed mechanism involves direct ischemia to the retrolaminar portion of the optic nerve head. Consistent with this hypothesis, the reduced vascularity of the choroid observed in NAION eyes may reflect susceptibility to ischemia. Overall, our results support multiple hypotheses relating to the pathogenesis of NAION, possibly reflecting the multifactorial nature of this enigmatic disorder.

PCT and CVI were negatively correlated among unaffected fellow eyes, but not among acute NAION eyes, non-acute NAION eyes, or controls. It is unclear whether this finding reflects drivers and/or consequences of ischemia in eyes at risk for NAION, but the relationship was robust and clearly restricted to unaffected fellow eyes. Longitudinal studies are needed to determine if the observed PCT/CVI relationship is a reliable marker for the development of sequential NAION. Perimetric performance was directly associated with vascular density only in non-acute NAION. A parsimonious interpretation of this finding is that greater vascularity may be protective and/or promote recovery following a NAION attack. It is unclear what the potential relationship between choroidal vascularity and perimetric performance in fellow eyes may indicate. We would speculate that susceptibility to ischemia may be reflected in subtle differences in perimetric performance among pre-clinical NAION eyes. Longitudinal studies evaluating choroidal vascularity and choroidal thickness in unaffected fellow eyes of NAION patients is needed to determine if CVI or PCT can predict sequential NAION.

Our study’s limitations include a relatively small number of usable NAION and fellow unaffected eyes due to SS-OCT imaging artifacts, segmentation error, and patient cooperation and fixation, all of which serve as barriers to obtaining high quality SS-OCT images. Additionally, acute and non-acute NAION were grouped together in this study given the limited sample of NAION cases, and peripapillary edema may alter PCT measurements in acute NAION. Furthermore, axial length and refractive error can influence OCT measures, but this information was not available for our NAION or control subjects. It is worth noting that the choroidal thickness measurement in our control group is similar to those reported elsewhere using SD-OCT and SS-OCT (3–6). Future prospective imaging studies should incorporate measurements of axial length and adjust accordingly to mitigate any potential confounding effects.

In conclusion, we show that NAION and unaffected fellow eyes demonstrate increased peripapillary choroidal thicknesses and reduced vascularity. Perimetric performance is directly associated with vascular density among non-acutely affected eyes with NAION. Longitudinal studies are needed to determine the clinical relevance of these structure-function relationships, both with regard to their applicability to neuro-ophthalmic practice and to provide further insights to the pathogenesis and pathophysiology of NAION.

## Figures and Tables

**TABLE 1 |:** Demographic, Ocular, and SS-OCT Characteristics Among NAION Eyes, Fellow Eyes, and Control Eyes.

	NAION Eyes (*n* = 17)	Fellow Eyes (*n* = 6)	Control Eyes (*n* = 18)	*P* Value, NAION *vs*. Control	*P* Value, Fellow *vs*. Control	*P* Value, NAION *vs*. Fellow

Age, years	62.8 ± 9.4	59.2 ± 9.0	60.5 ± 5.5	>0.99	>0.99	>0.99
Sex, male (%)	13 (76.5)	6 (100)	13 (72.2)	>0.99	0.50	0.73
BCVA, logMAR	0.12 ± 0.3	−0.02 ± 0.1	0.02 ± 0.1	0.30	>0.99	0.35
IOP, mm Hg	15.0 ±2.2	15.6 ± 2.9	15.8 ± 3.0	>0.99	>0.99	>0.99
HVF mean deviation, dB	−13.1 ± 7.3	−0.5 ± 1.5	N/A	N/A	N/A	<0.001*
HVF pattern standard deviation, dB	11.7 ± 4.7	1.5 ± 0.3	N/A	N/A	N/A	<0.001*
Mean PCT, µm	278.0 ± 65.2	221.4 ± 50.4	157.5 ± 26.9	<0.001*	0.028*	0.063
Superior quadrant PCT, µm	278.8 ± 77.0	215.6 ± 61.7	150.2 ± 27.0	<0.001*	0.063	0.08
Inferior quadrant PCT, µm	263.3 ± 90.5	205.5 ± 58.9	135.8 ± 36.2	<0.001*	0.10	0.23
Nasal quadrant PCT, µm	278.1 ± 58.8	226.1 ± 51.1	175.9 ± 27.7	<0.001*	0.082	0.069
Temporal quadrant PCT, µm	291.7 ± 76.0	238.6 ± 50.8	167.9 ± 33.5	<0.001*	0.038*	0.173
Mean CVI	0.35 ± 0.03	0.35 ± 0.04	0.38 ± 0.02	<0.008*	0.068	>0.99
Nasal CVI	0.38 ± 0.03	0.38 ± 0.02	0.40 ± 0.02	0.035*	0.65	>0.99
Temporal CVI	0.33 ± 0.04	0.32 ± 0.06	0.37 ± 0.04	0.048*	0.095	>0.99

SS-OCT, swept-source optical coherence tomography; NAION, non-arteritic anterior ischemic optic neuropathy; BCVA, best-corrected visual acuity; logMAR, logarithm of the minimum angle of resolution; IOP, intraocular pressure; HVF, Humphrey visual field; PCT, peripapillary choroidal thickness; CVI, choroidal vascularity index.

Values presented as mean ± standard deviation unless otherwise noted.

* indicates *P*<0.05, Bonferroni-corrected pairwise comparisons.

**TABLE 2 |:** Previous OCT studies investigating the peripapillary choroid in NAION.

Study (first author, year)	*n*, NAION eyes	OCT Name	OCT Type	PCT*	CVI*	Identified Visual Correlates?

García-Basterra (2016) (4)	37 (37 non-acute, 19 fellow, 38 control)	Cirrus	SD-OCT	↓	N/A	No
Fard (2015) (5)	30 (30 non-acute, 30 fellow, 25 control)	Spectralis	SD-OCT	↑	N/A	No
Nagia (2016) (3)	20 (20 non-acute, 10 fellow, 102 control)	Spectralis	SD-OCT	↑	N/A	No
Jiang (2016) (8)	44 (19 acute, 25 non-acute, 44 fellow, 60 control)	Spectralis	SD-OCT	↔	N/A	No
Nikkhah (2020) (9)	38 (38 acute, 38 fellow, 74 control)	Spectralis	SD-OCT	↑	N/A	No
Perez-Sarriegui (2018) (6)	29 (29 non-acute, 21 fellow, 29 control)	Triton	SS-OCT	↑	N/A	No
Guduru (2019) (7)	20 (20 acute, 20 fellow, 40 control)	Triton	SS-OCT	N/A	4	No
Current Study	17 (9 acute, 8 non-acute, 6 fellow, 18 control)	Triton	SS-OCT	↑	4	Yes

PCT, peripapillary choroidal thickness; CVI, choroidal vascularity index; NAION, non-arteritic anterior ischemic optic neuropathy; SD-OCT, spectral-domain optical coherence tomography; SS-OCT, swept-source optical coherence tomography.

* NAION compared to controls (↑, Increased; ↓, decreased; ↔, no difference; N/A, did not assess).

## Data Availability

The raw data supporting the conclusions of this article will be made available by the authors, without undue reservation.
